# 12/15-Lipoxygenase-Derived Lipid Metabolites Induce Retinal Endothelial Cell Barrier Dysfunction: Contribution of NADPH Oxidase

**DOI:** 10.1371/journal.pone.0057254

**Published:** 2013-02-20

**Authors:** Amira Othman, Saif Ahmad, Sylvia Megyerdi, Rene Mussell, Karishma Choksi, Krishna Rao Maddipati, Ahmed Elmarakby, Nasser Rizk, Mohamed Al-Shabrawey

**Affiliations:** 1 Department of Oral Biology/Anatomy, College of Dental Medicine, Georgia Regents University (GRU), Augusta, Georgia, United States of America; 2 Department of Anatomy, Mansoura University, Mansoura, Egypt; 3 Department of Ophthalmology and Vision Discovery Institute, Medical College of Georgia, Georgia Regents University (GRU), Augusta, Georgia, United States of America; 4 Department of Pathology, Wayne States University, Detroit, Michigan, United States of America; 5 Vascular Biology Center, Medical College of Georgia, Georgia Regents University (GRU), Augusta, Georgia, United States of America; 6 Department of Health Sciences, College of Science, Qatar University, Doha, Qatar; University Heart Centre Freiburg, Germany

## Abstract

The purpose of the current study was to evaluate the effect of 12/15- lipoxygenase (12/15-LOX) metabolites on retinal endothelial cell (REC) barrier function. FITC-dextran flux across the REC monolayers and electrical cell-substrate impedance sensing (ECIS) were used to evaluate the effect of 12- and 15-hydroxyeicosatetreanoic acids (HETE) on REC permeability and transcellular electrical resistance (TER). Effect of 12- or 15-HETE on the levels of zonula occludens protein 1 (ZO-1), reactive oxygen species (ROS), NOX2, pVEGF-R2 and pSHP1 was examined in the presence or absence of inhibitors of NADPH oxidase. *In vivo* studies were performed using Ins2^Akita^ mice treated with or without the 12/15-LOX inhibitor baicalein. Levels of HETE and inflammatory mediators were examined by LC/MS and Multiplex Immunoassay respectively. ROS generation and NOX2 expression were also measured in mice retinas. 12- and 15- HETE significantly increased permeability and reduced TER and ZO-1expression in REC. VEGF-R2 inhibitor reduced the permeability effect of 12-HETE. Treatment of REC with HETE also increased ROS generation and expression of NOX2 and pVEGF-R2 and decreased pSHP1 expression. Treatment of diabetic mice with baicalein significantly decreased retinal HETE, ICAM-1, VCAM-1, IL-6, ROS generation, and NOX2 expression. Baicalein also reduced pVEGF-R2 while restored pSHP1 levels in diabetic retina. Our findings suggest that 12/15-LOX contributes to vascular hyperpermeability during DR via NADPH oxidase dependent mechanism which involves suppression of protein tyrosine phosphatase and activation of VEGF-R2 signal pathway.

## Introduction

Diabetic retinopathy (DR) is the most common cause of blindness in working age Americans. The presence of an intact blood–retinal barrier (BRB) is essential for retinal structural and functional integrity. Vision is adversely affected in clinical conditions associated with the breakdown of BRB such as DR or age related macular degeneration (AMD). Development of DR begins with early inflammatory response as shown by early onset of increased leukostasis and vascular permeability. Retinal inflammation is followed by capillary degeneration, ischemia, and finally uncontrolled neovascularization to compensate for the lack of blood flow [1], [2], [3].

In addition to persistent hyperglycemia, dyslipidemia was reported to contribute to microvascular dysfunction during DR [4], [5], [6]. However, its role in the development of retinal microvascular complications has not been studied in detail [6]. Diabetic dyslipidemia is characterized by an increase in n-6 polyunsaturated fatty acids (PUFA), such as arachidonic acid (AA) [7] which is released from the cell membrane by cytosolic phospholipase A_2_ (cPLA_2_). Arachidonic acid is considered a target for different enzymatic pathways such as cycloxygenase (COX2), lipoxygenase (LOX), and cytochrome P450 (CYP). [8], [9] Lipoxygenases are a group of closely related dioxygenases that are classified as 5-, 8-, 12-, or 15-LOX, according to the site of oxygen insertion within AA. [10]. 12/15-LOX pathway has proven to be involved in cardiovascular complications of diabetes such diabetic nephropathy, atherosclerosis and hypertension [11], [12], [13], [14]. The early inflammatory reaction in DR such as leukostasis has been correlated to the LOX pathways [6], [15], [16]. Moreover, we recently demonstrated that pathological retinal neovascularization (NV) in humans with proliferative diabetic retinopathy (PDR) and mouse model of oxygen-induced retinopathy (OIR) was associated with significant increase in LOX-derived eicosanoids, 12-, 15- and 5- hydroxyeicosatetreanoic acids (HETE) [10]. Additionally, pharmacological inhibition or deletion of 12/15-LOX led to marked reduction in retinal NV in OIR [10] suggesting that lipoxygenase pathways in general and 12/15-LOX in particular play a key role in the development of microvascular dysfunction during DR. The current study extends our previous findings and focuses on the role of 12/15-LOX in vascular hyperpermeability during DR. Recently, baicalein a known pharmacological inhibitor of 12/15-LOX was shown to prevent the early microvascular dysfunction and inflammatory response in rat model of experimental diabetes [17].

Oxidative stress has been correlated to diabetes-induced microvascular inflammatory reactions and dysfunction [18]. Increased activity of NADPH oxidase in diabetic patients, animals, and high glucose-treated endothelial cells has been shown in previous studies [18], [19], [20], [21] suggesting that NADPH oxidase is an important source of reactive oxygen species (ROS). We and others showed that endothelial NADPH oxidase plays a crucial role in causing vascular inflammation and leakage in models of DR [22], [23], [24] as well as retinal NV [25].

The goal of the current study was to test the hypothesis that 12/15-LOX contributes to vascular hyperpermeability during DR via the activation of NADPH oxidase. For this purpose, we evaluated the direct effect of 12/15-LOX metabolites on endothelial cell barrier function in the presence or absence of NADPH oxidase inhibitors. We also tested the impact of inhibiting 12/15-LOX on the levels of tight junction protein (TJP), cytokines and ROS generation in retina of diabetic mice. Our findings suggest that activation of 12/15-LOX is a contributing factor to the vascular hyperpermeability during DR and that NADPH oxidase plays a role in this process via activating VEGF-R2 signal pathway.

## Materials and Methods

### Ethics Statement

All animal experiments followed the guidelines established by the Association for Research in Vision and Ophthalmology (ARVO) Statement for the Use of Animals in Ophthalmic and Vision Research. The protocol was approved by the Institutional Animal Care and Use Committee (IACUC) of the Georgia Health Sciences University. Mice were sacrificed using carbon dioxide (CO2) inhalation and all efforts were made to minimize suffering.

### Experimental mice

Ins2^Akita^ mice of C57BL/6J background (Jackson Lab, Bar Harbor, ME) were used as an animal model of retinal complications in diabetes [26]. Heterozygous Ins2^Akita^ mice were bred to C57BL/6J mice, and male offspring were monitored for hyperglycemia, beginning at 4 weeks of age. These mice develop hyperglycemia by 4 weeks of age and also develop retinal changes similar to DR such as vascular leakage, neuronal cell death and leukostasis. Mice with glucose level >250 mg/dl for 10–12 weeks were used for our experiments as diabetic group. One group of diabetic mice received the 12/15-LOX inhibitor baicalein at a dose of 75 mg/kg/day in drinking water (Cayman Chemical, Ann Arbor, Michigan) starting from the onset of diabetes. We used 5–8 mice from each group for each set of our experiments.

### Microvascular retinal endothelial cells

*In vitro* experiments were performed using primary culture of bovine retinal endothelial cells (BRECs) which were isolated from bovine retinas following an established protocol [27]. The cells (passages 6–8) were grown on gelatin-coated dishes and maintained in M199 media supplemented with 4.5 mM glucose, 2 mM L-glutamine, CSC growth factor penicillin/streptomycin, and 10% FBS. After the cells formed complete confluent layer, the cells were shifted to 1% fetal bovine serum overnight, then treated with 0.1 µM of 12- or 15-HETE (Cayman Chemical, Ann Arbor, MI) with or without apocynin 30 µM, diphenylene iodonium (DPI, 5 µM) or N-acetyl-L-cystein (NAC) 50 µM (Sigma-Aldrich). Experiment was terminated after 24 hours, then cells were homogenized with RIPA buffer and cell lysate was collected for further immunoblotting assay.

### Assessment of retinal endothelial cell barrier function

#### FITC-Dextran permeability assay

Retinal endothelial cells (REC) were seeded on collagen/fibronectin coated membranes with 0.4 µm pores (Transwell; Corning Costar), in normal glucose media. After becoming completely confluent cells were shifted to 1% serum media overnight then were treated by 0.1 µM 12- or 15-HETE in the presence or absence of apocynin (30 µM), DPI (5 µM) or NAC (50 µM) in the upper chambers for 12 hours. VEGF, 100 ng/ml (Shenandoah Biotechnology) was used as positive control. FITC-dextran (1 mg/ml) was then added to the upper chambers followed by obtaining aliquots from the lower and upper chambers at different time points (0.5, 1, 4 or 6 hrs) then measuring the fluorescence intensity with a plate reader. The FITC-dextran that passed across the REC monolayer was corrected to the fluorescence reading of samples from the upper chamber.

Additional experiment was performed in which REC were treated with 12-HETE in the presence or absence of 10 nM of the relatively selective VEGF-R2 inhibitor ZM323881 hydrochloride (R&D System Minneapolis, MN). FITC-Dextran leakage then was measured over 4 hours. ZM323881 is known to inhibit VEGF-induced endothelial cell proliferation and permeability through inhibiting the VEGF-R2 tyrosine phosphorylation [28], [29], [30], [31].

### Measurement of transendothelial electrical resistance (TER)

Retinal endothelial cells were seeded at a density of 5×10^4^ cells/well with gold electrodes (96W10E+), each was coated with cystein, collagen and fibronectin. The electric currents passing through fully confluent monolayers are measured independently in each well by the Electrical Cell–Substrate Impedance Sensing (ECIS from Applied Biophysic, Inc., Troy, NY.) Cells were starved for 24 hours and then treated with or without 12- or 15-HETE (0.1 µM) and VEGF 100 ng/ml. TER was recorded over the experimental time course (24 hours). Resistance values for each chamber were normalized as the ratio of measured resistance at each time point (15 min) to baseline resistance (normalized resistance) and plotted as a function of time.

### Measurement of 12/15-LOX -derived eicosanoids

Activity of the 12/15-LOX was evaluated by LC/MS to measure the amount of HETE in retina as described by us before [10]. Briefly, samples were spiked with 10 ng of 15(S)-HETE-d_8_ (internal standard), acidified to pH <4 with dilute hydrochloric acid, applied to preconditioned SEP-Pak C18 cartridges (100 mg adsorbent, Waters), washed with water followed by hexane. Eicosanoids were eluted with 500 µl of ethyl acetate, then dried under nitrogen and reconstituted in methanol:25 mM aqueous ammonium acetate (8 2). This was followed by subjecting the extracted and reconstituted sample to HPLC on a Max-RP C18 column (2×150 mm, 3 µ, Phenomenex). Methanol:13 mM aqueous ammonium acetate (8 2) at a flow rate of 0.4 ml/min was used to elute the compounds isocratically. Then, HETE were monitored by mass spectrometer (QuattroLC, Micromass) in the negative ion mode using Multiple Reaction Monitoring for transitions of m/z 319 to m/z 115 for 5-HETE, m/z 179 for 12-HETE, and m/z 219 for 15-HETE (Source block: 120 °C, Desolvation: 350 °C, Cone voltage: -24 V, Collision energy: 14 eV, and Collision gas pressure: 3.2×10^-4^). 15(S)-HETE-d_8_ (MRM transition of m/z 327 to 226 under identical conditions) was used as an internal standard for recovery and quantitation. Under these conditions, retention times for 15-HETE (and 15(S)-HETE-d_8_), 12-HETE, and 5-HETE were 2.6, 2.9, and 3.6 min, respectively and the detection limit was 50 pg for each compound on the column.

### Cytokines and adhesion molecules assay

Levels of inflammatory mediators in mouse retina were measured by Multiplex System. Retinas were extracted from the mice eyes, snap frozen in liquid nitrogen and then shipped to the Human Bioscience, Inc. (Gaithersburg, MD) for multiplex analysis of the levels of retinal ICAM-1, VCAM-1, and IL-6 in different experimental mouse groups.

### Measurement of ROS generation

#### Superoxide measurement by dihydroethidium (DHE)

Superoxide generation causes oxidation of DHE into ethidium bromide, which binds to DNA in the nucleus and fluoresces red [32]. BRECs were treated by 12-HETE in the presence or absence of NADPH (100 µM) with or without apocynin (30 µM) or superoxide dismutase (PEG-SOD, 400 U). The medium was then replaced by Earle’s balanced salt solution (EBSS) containing the same inhibitors and DHE (2 µM) followed by incubation for 20 minutes in water bath (37°C). Images were then collected by fluorescence microscope and analyzed by Metamorph Imaging System (Universal Imaging Corporation, Downingtown, PA) for fluorescence intensity in equal number of cells (10 cells/field, five fields total). Similarly DHE staining was used to assess superoxide generation in fresh retinal sections from different mouse groups.

### Intracellular ROS measurement by dichlorofluorescein (DCF)

OxiSelect ROS Assay Kit (Cell Biolabs, San Diego, CA) was used to measure ROS activity. The assay utilizes the diffusion of 2’,7’-Dichlorodihydrofluorescin diacetate (DCFH-DA) into cells thereby becoming deacetylated to DCFH and rapidly oxidized to DCF by ROS. In brief, RECs were cultured in 96-well plates and preincubated with DCFH-DA and NADPH (100 µM) for 20 minutes before adding 12-HETE with or without apocynin or PEG-SOD for one hour. A standard fluorescent plate reader was used to determine ROS production by comparing results with a predetermined DCF standard curve.

### Western blotting

Western blotting was used to assess the expression of NOX2, ZO-1, ICAM-1, pVEGF-R2 VEGF-R2, and pSHP1 in BRECs lysate or mouse retinal homogenate from different experimental groups. Retinal samples were homogenized in a modified RIPA buffer (20 mM Tris-HCl [33], 2.5 mM ethylenediaminetetraacetic acid, 50 mM NaF, 10 mM Na_4_P_2_O_7_, 1% Triton X-100, 0.1% sodium dodecyl sulfate, 1% sodium deoxycholate, 1 mM phenylmethylsulfonyl fluoride). Homogenates (30–50 µg protein) were separated by electrophoresis on a precast Tris-HCl 4–20% gradient gel, and transferred to nitrocellulose membrane. Retina homogenates were reacted with primary antibodies against NOX2 or gp91^phox^ (mouse monoclonal, 1 500, BD transduction laboratories, San Diego, CA), ZO-1, 2 µg/ml (Invitrogen, Eugene, OR), ICAM-1 (1 250, Santa Cruz, Santa Cruz, CA), pVEGF-R2, VEGF-R2 (1 250, Abcam, Cambridge, MA) and pSHP1 (1 200, Stem Cell Technologies, Vancouver, Canada). Protein was detected by utilizing horseradish peroxidase-linked secondary antibodies and enhanced chemiluminescence (Amersham, Pittsburgh, PA). Membranes were stripped and re-probed for β-actin 1 2000 (Abcam, Cambridge, MA) to demonstrate equal loading and the results were quantified by densitometry analysis.

### Immunofluorescence

The expression of ZO-1 was colocalized with retinal vasculatures which were labeled red with DyLight 594 labeled Lycopersicon Esculentum Lectin (LEL, TL) (Vector, Burlingame, CA). Eye balls were removed then frozen sections were prepared at 10 µm thick sections. Sections were fixed with 4% formalin for 10 minutes then incubated with ZO-1 (1 100) antibody at 4°C overnight followed by blocking non specific reaction using DAKO blocking buffer (DAKO, Carpinteria, CA). Retinal sections then were incubated in Oregon green labeled secondary antibody1 500 (Molecular Probes, Eugene, OR) mixed with Lectin 5 µl/ml, washed then covered using anti-fad mounting medium and DAPI as a nuclear marker. Images were collected and color intensity was measured by Metamorph Imaging System. Similarly, NOX2 imunoreactivity was performed using a specific NOX2 antibody.

ZO-1 immunoreactivity was also performed in BRECs treated with or without 12- or 15-HETE in the presence or absence of NAC, apocynin or DPI. We used Lab-Tek II chamber slides (NUNC, Rochester, NY) where 70×10^3^ cells were seeded and incubated for 24 hrs in complete serum before switching to serum free medium. This was followed by adding the aforementioned treatment. Cells then were fixed with 2% formalin for 10 minutes and blocked with normal goat serum for one hr followed by incubation with ZO-1 antibody (1 100) for 2 hrs at room temperature before incubation with Oregon green labeled secondary antibody (1 500, Invitrogen, Eugene, OR). Images then were collected and color intensity was measured by Metamorph Imaging System.

We also evaluated the impact of baicalein treatment on the levels of pVEGF-R2 and pSHP1in retinas of diabetic mice. For this purpose retinal sections from different experimental groups were incubate with pVEGF-R2 or pSHP1 (1 100 each) antibody and isloectin B4 as a vascular marker followed by the Oregon green labeled secondary antibody and Texas Red to visualize the pVEGF-R2 or pSHP1 and the isolectin B4 respectively. Images were collected and color intensity was measured as above.

### Statistical Analysis

Group differences were evaluated using ANOVA followed by Tukey’s post hoc test. t-test was used if needed to detect any significant difference between two groups. Results were considered significant when P value <0.05. For *in vivo* studies, age matched control mice were compared with diabetic mice treated with or without baicalein. For *in vitro* studies, at least 4 dishes were prepared for each treatment group and each experiment was replicated with at least 3 different batches of retinal cells. Data was represented as mean +SE from at least 5 animals in each group and 3 experiments from the *in vitro* study.

## Results

### Effect of 12- and 15-HETE on retinal endothelial cell barrier

**Figure 1:**
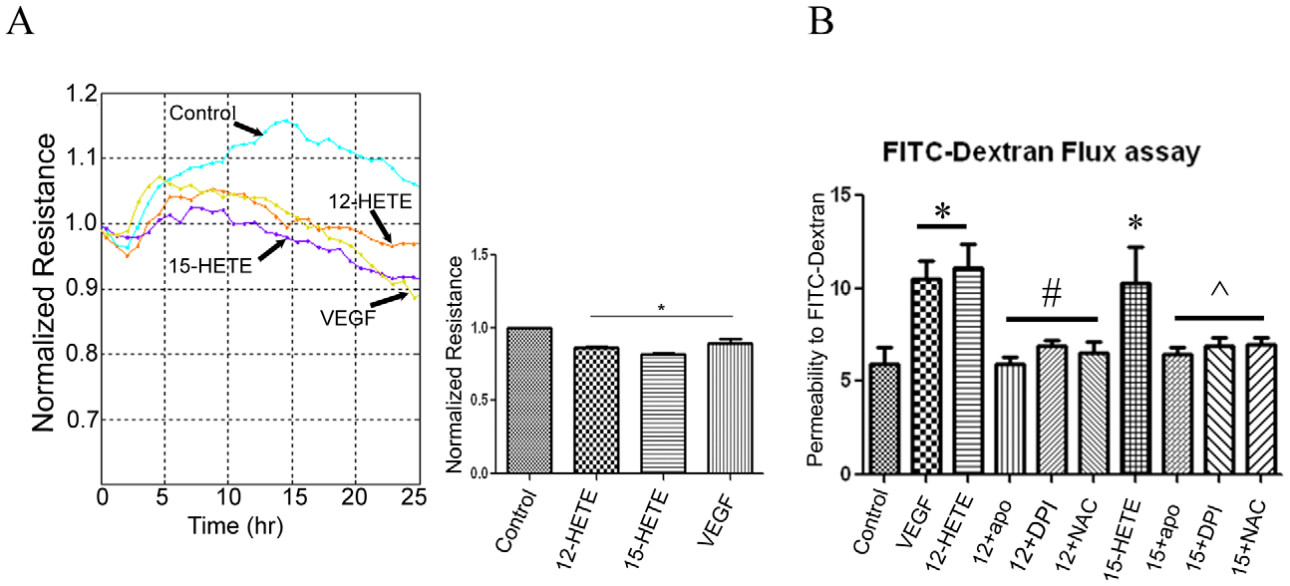
Effect of HETE on REC barrier function. Measurement of transcellular electrical resistance (TER)in REC (A) showed significant decreases in the TER by VEGF, 12- and 15-HETE, compared to the control (* P<0.05 vs control, n = 3). Assessment of REC permeability by FITC-dextran flux method (B). Retinal endothelial cells were incubated with VEGF (100 ng/ml) 12-HETE or 15-HETE (0.1 µM) for 12 hrs before adding FITC-Dextran to the top chamber of the transwell. Four hours later fluorescence intensity in the medium of the lower chamber was measured by a plate reader and normalized to the fluorescence intensity in the upper chamber. *There was significant increase in the FITC-dextran leakage by VEGF, 12- and 15-HETE in comparison to the control. Effect of 12- and 15-HETE on FITC-dextran leakage was blocked by the NADPH oxidase inhibitors (apocynin and DPI), and the antioxidant N-acetyl-L- cystein (NAC). *P<0.05 vs control, # P<0.05 vs 12-HETE, ∧ P<0.05 vs 15-HETE (n = 7).

To assess the effect of 12- and 15-HETE on REC barrier function, we examined whether 12- or 15-HETE induces changes in FITC dextran flux and TER in BREC confluent monolayer. Measurement of TER demonstrated significant decrease by 12- and 15- HETE compared to the control (Fig. 1A). Effect of 12- and 15-HETE on the TER was comparable to the effect of VEGF. Changes in TER was first observed after 4 hrs of treatment with HETE and continued to decrease through the experiment. We also determined the effect of the 12/15-LOX metabolites on REC barrier function by measuring FITC-dextran leakage through REC confluent monolayer. We noticed that REC became significantly permeable to FITC-dextran when treated with VEGF, 12- or 15-HETE for 12 hrs. The maximum leakage was noticed after 4 hrs from adding the FITC-dextran (Fig. 1B). To evaluate whether NADPH oxidase plays a role in mediating the pro-permeability effect of 12- or 15-HETE, we tested the effect of two NADPH oxidase inhibitors, apocynin and DPI on HETE-induced permeability in comparison to the ROS scavenger NAC. Apocynin, DPI and NAC blocked the effect of 12- and 15-HETE on FITC-dextran leakage (Fig. 1B).

### Effect of 12- and 15-HETE on ZO-1 expression in REC

**Figure 2:**
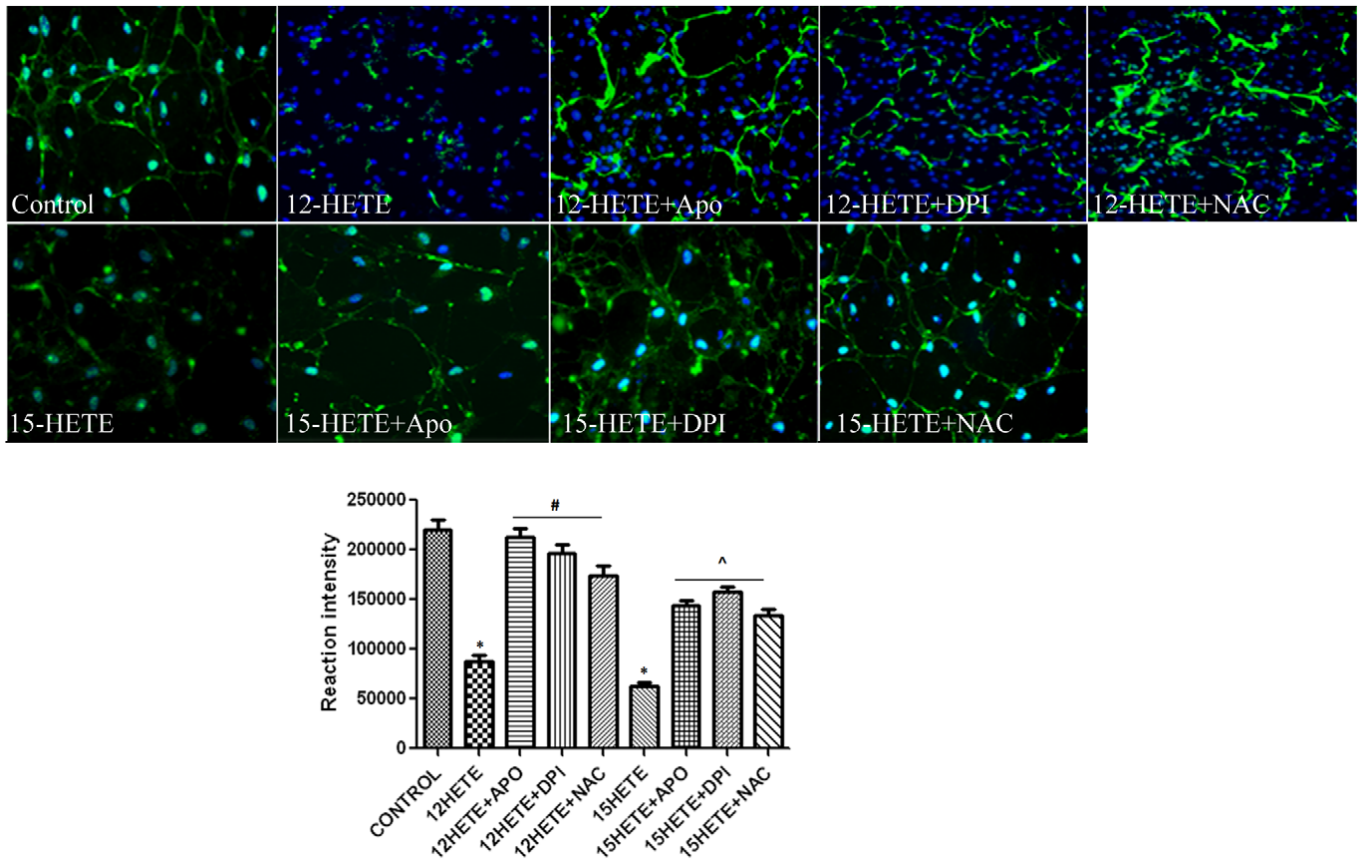
Effect of HETE on ZO-1 expression in retinal endothelial cells. Immunofluorescence of ZO-1 (green) in REC treated by 12- or 15-HETE. There was marked decrease in ZO-1 expression by 12- and 15-HETE. Effect of 12- and15-HETE on ZO-1 expression in the REC was prevented by apocynin, DPI and NAC. DAPI (blue color) is a nuclear marker *P<0.05 vs control, # P<0.05 vs 12- or 15-HETE (n = 6).

Disruption of TJPs is crucial for breakdown of the BRB. Therefore, we examined if 12/15-LOX -derived HETE modulate expression of ZO-1, a tight junction intracellular protein in REC. 12- and 15-HETE (Fig. 2) elicited remarkable decrease in ZO-1 expression compared to the control. Effect of HETE on ZO-1 expression was blocked by apocynin, DPI and NAC.

### Effect of baicalein treatment on HETE production in mouse retina

**Figure 3:**
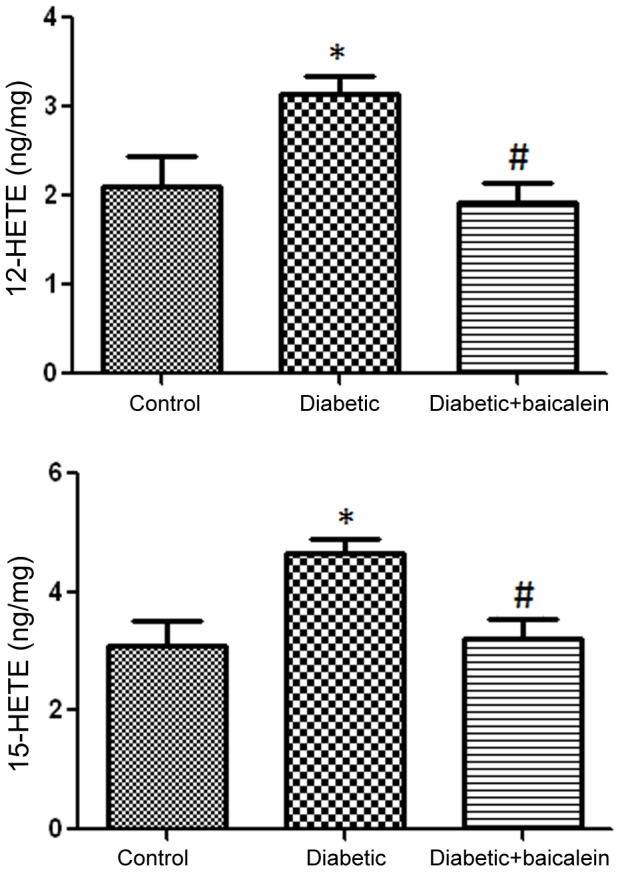
LC/MS assay of retinal HETE. There was significant increase in retinal 12- and 15-HETE in diabetic mice compared to the control. Effect of diabetes on HETE generation was significantly reduced by the 12/15LOX pharmacological inhibitor baicalein. *P<0.05 vs Control, # P<0.05 vs diabetic (n = 5).

Because we previously demonstrated that retinal 12/15-LOX was upregulated in retina of diabetic mice [10] we further evaluated 12/15-LOX activity by measuring the levels of its lipid metabolites in different experimental groups. We noticed a significant increase in the amount of 12- and 15-HETE in retina of diabetic mice compared to the control. This increase in the levels of 12- or 15-HETE was prevented in mice treated with baicalein supporting the rational that baicalein targets 12/15-LOX activity (Fig. 3).

### Effect of baicalein treatment on the inflammatory mediators in diabetic retina

**Figure 4:**
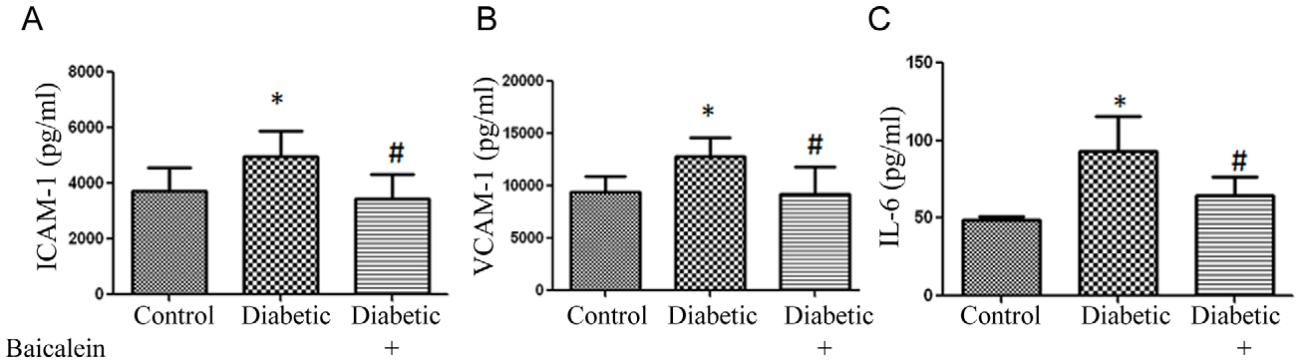
Effect of 12/15-LOX inhibition on the levels of inflammatory mediators in retina of diabetic mice. Analysis of IL-6 and adhesion molecules expression in mouse retina using multiplex system showed significant increase in the levels of ICAM-1 (A), VCAM-1 (B) and IL-6 (C) in diabetic group compared to the control. These increases were prevented in baicalein treated group. *P<0.05 vs Control, # P<0.05 vs Diabetic (n = 7).

Our previous studies demonstrated that IL-6 and adhesion molecules are important factors in mediating angiotensin as well as diabetes-induced retinal vascular injury [23], [34], [35]. Furthermore, the aqueous humor cytokine profile revealed that compared to diabetic patients without retinopathy, only the IL-6 and VEGF levels were significantly higher in diabetic patients with retinopathy [36]. Therefore, to test whether 12/15-LOX -derived HETE are involved in the early inflammatory response during DR, we examined the effect of baicalein on the inflammatory mediators, IL-6, ICAM-1 and VCAM-1 (Fig. 4). There was a significant increase of ICAM-1 and VCAM-1 in the diabetic group compared to the control. Effect of diabetes on ICAM-1 and VCAM-1 was significantly blocked by baicalein. There was also marked increase in retinal level of IL-6 in diabetic mice which was also blocked by baicalein.

### Effect of baicalein treatment on ZO-1 expression in mouse retina

**Figure 5:**
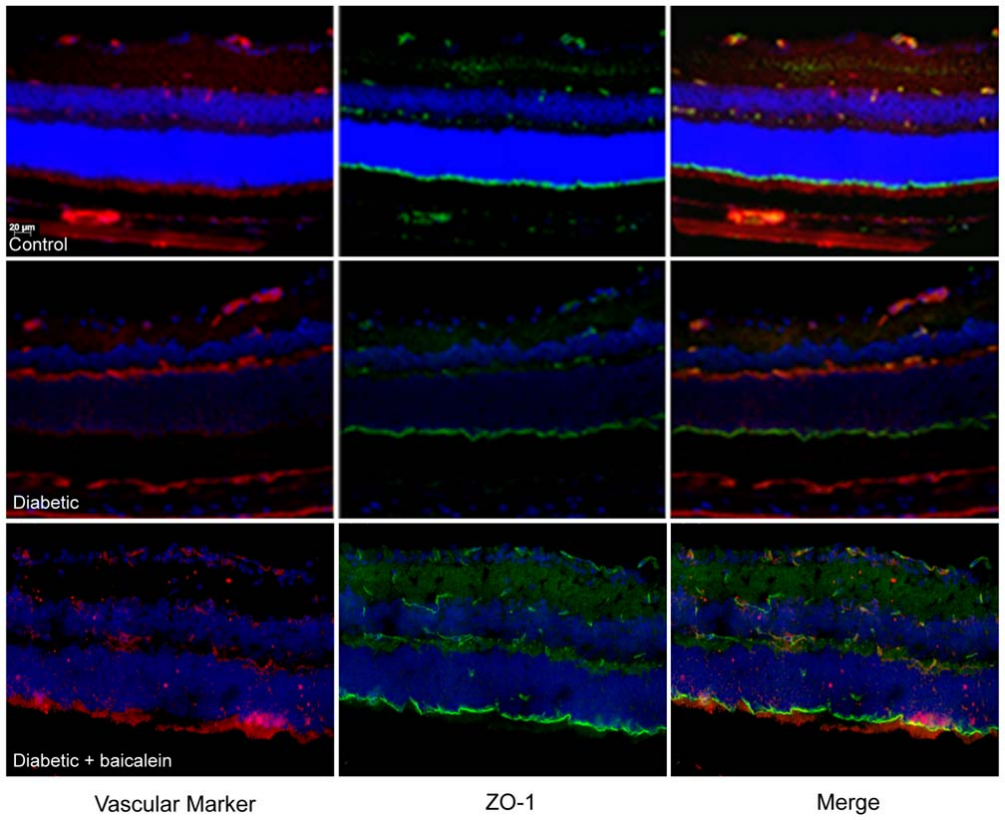
ZO-1 Immunoreactivity in retinal sections. Co-localization of ZO-1 (green), retinal vessels (red) and nuclear staining DAPI (blue) showed marked reduction in ZO-1 expression by diabetes compared to the control. Note, restoration of the ZO-1 expression in retina of baicalein- treated mice. *P<0.05 vs Control, # P<0.05 vs Diabetic (n = 8).

Because baicalein has shown previously to attenuate retinal vascular permeability in diabetic rats [17] we also assessed the impact of baicalein on the levels of the tight junction protein ZO-1. Our experiments showed restoration of retinal expression of ZO-1 in diabetic mice by baicalein to normal level (Fig. 5).

### 12-HETE modulates NADPH oxidase activity and NOX2 expression in REC

**Figure 6:**
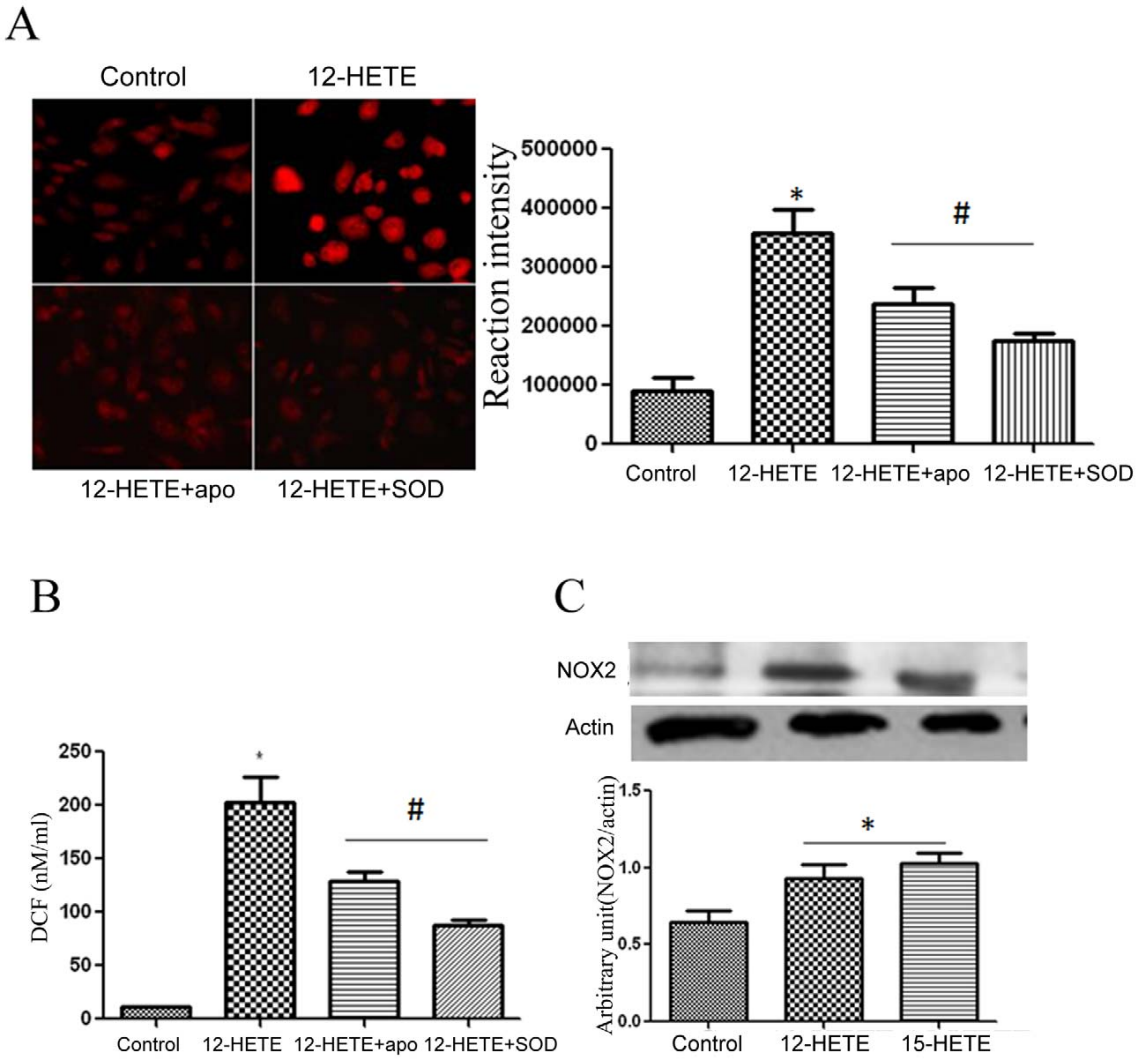
Effect of HETE on ROS generation and expression NOX2 in retinal endothelial cells. DHE staining (A) and DCF assay (B) showed marked increase in ROS generation in REC by 12-HETE. Note significant reduction of 12-HETE-induced ROS generation by apocynin and SOD. Western blotting analysis of NOX2 expression (C) in REC showed upregulation by 12- and 15-HETE compared to the control. *P<0.05 vs Control, # P<0.05 vs 12-HETE (n = 6).

To determine whether HETE are upstream of ROS generation in diabetic retina, we evaluated the effect of HETE on ROS generation in BRECs using DHE staining and DCF assay methods. There was significant increase in ROS generation by 12-HETE which was blocked by PEG-SOD and the NADPH oxidase inhibitor apocynin (Fig. 6A and B) suggesting that NADPH oxidase is an important source of 12/15-LOX -induced oxidative stress. Furthermore, treatment of REC with 12- or 15-HETE significantly increased NOX2 expression compared to the control (Fig. 6C).

### Baicalein reduced ROS generation and NOX2 expression in diabetic retina

**Figure 7:**
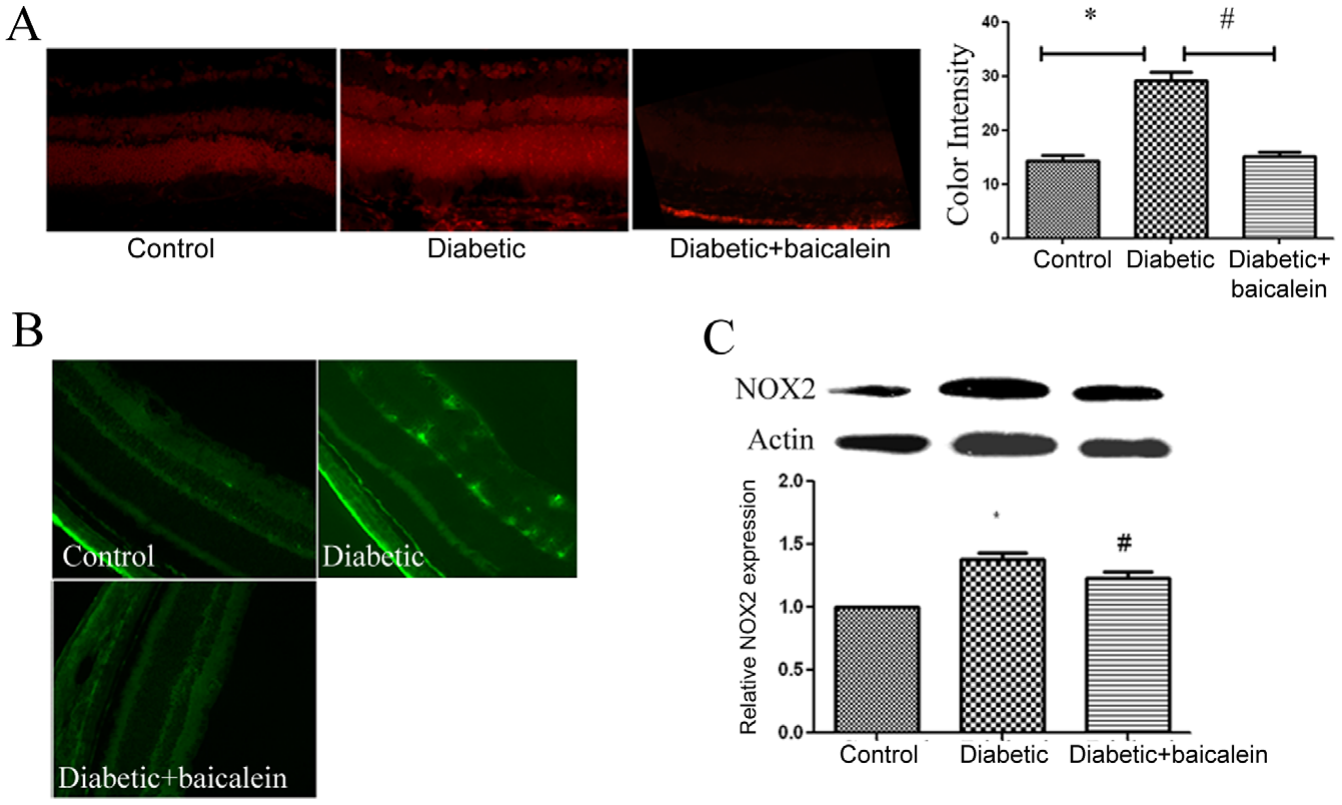
Effect of baicalein on ROS generation and NOX2 expression in diabetic retina. DHE staining (A) of retinal section showed marked increase in ROS generation in diabetic mice which was prevented by baicalein. Immunofluorescence (B) and Western blot analysis of NOX2 (C) in mouse retina demonstrated marked upregulation by diabetes and this increase was significantly reduced by baicalein. *P<0.05 vs C, # P<0.05 vs D (n = 6).

Because we previously showed that NADPH oxidase-derived ROS play a role in vascular hyperpermeability during DR [22], [23], we also determined whether NADPH oxidase is involved in 12/15-LOX -induced vascular changes during DR. Inhibiting 12/15-LOX by baicalein reduced diabetes-induced ROS generation and NOX2 expression in mouse retina (Fig. 7).

### Effect of 12-HETE on VEGF-R2 and SHP-1 in BRECs

**Figure 8:**
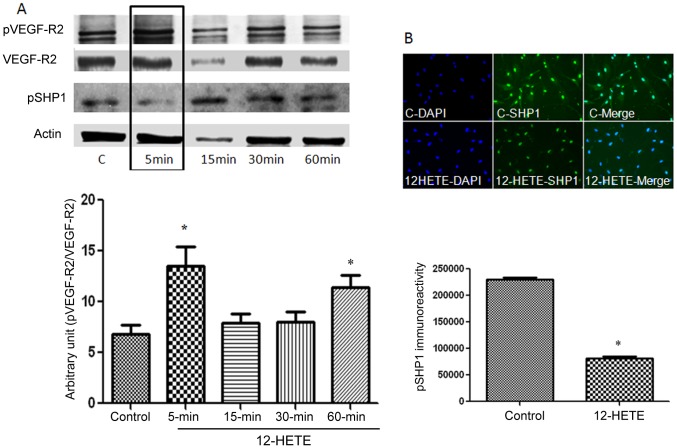
12-HETE induces phosphorylation of VEGF-R2 and dephosphorylation of protein tyrosine phosphatase (PTP) SHP1 in REC. Western blotting analysis (A) demonstrated significant increase in the level of pVEGF-R2 after 5 and 60 minutes from the beginning of treatment with 12-HETE. *P<0.05 vs Control (C) and other time points. There was simultaneous decrease in the level of pSHP1. Immunofluorescence of pSHP1 (green) in REC also showed marked decrease in the pSHP1 immunoreactivity by 12-HETE compared to the control. * P<0.05 vs control (n = 3).

Our previous study showed that 12-HETE modulates VEGF expression in retinal glial cells. Accordingly, we tested whether 12-HETE also impact VEGF-R2 in REC. Our experiments showed that 12-HETE induced phosphorylation of VEGF-R2 and simultaneous dephosphorylation of the protein tyrosine phosphatase SHP-1 (Fig. 8). Therefore, in addition to the paracrine effect on VEGF expression in glial cells, endothelial cell-derived HETE may also elicit an autocrine effect via inducing REC VEGF-R2 signaling pathway.

### Effect of VEGF-R2 inhibition on 12-HETE-induced REC hyperpermeability

**Figure 9:**
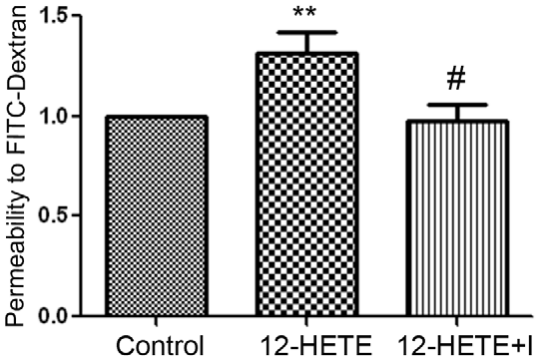
Effect of VEGF-R2 inhibition on 12-HETE-induced REC hyperpermeability. Retinal endothelial cells were treated with 0.1 µM 12-HETE in the presence or absence of the VEGF-R2 inhibitor, ZM323881 hydrochloride (10 nM) for 12 hrs before adding the FITC-dextran to the upper chamber of the transwell. Four hrs later the fluorescence intensity in the lower chamber was measured by the plate reader and corrected to the intensity in the upper one. The permeability effect of 12-HETE was significantly prevented by the ZM323881 hydrochloride (12-HETE+I). * P<0.05 vs control and # P<0.05 vs 12-HETE (n = 5).

To test whether activation of VEGF-R2 is involved in the REC hyperpermeability induced by the 12/15-LOX lipid metabolites we tested the effect of 12-HETE on REC barrier function in the presence or absence of the relatively selective VEGF-R2 inhibitor ZM323881 hydrochloride. While there was 1.3 fold increase in REC permeability by 12-HETE after 4 hrs compared with the non-treated cells, this increase was inhibited by the VEGF-R2 inhibitor ZM323881 (Fig. 9).

### Baicalein reduced pVEGF-R2 and restored pSHP1 levels in retinas of diabetic mice

**Figure 10:**
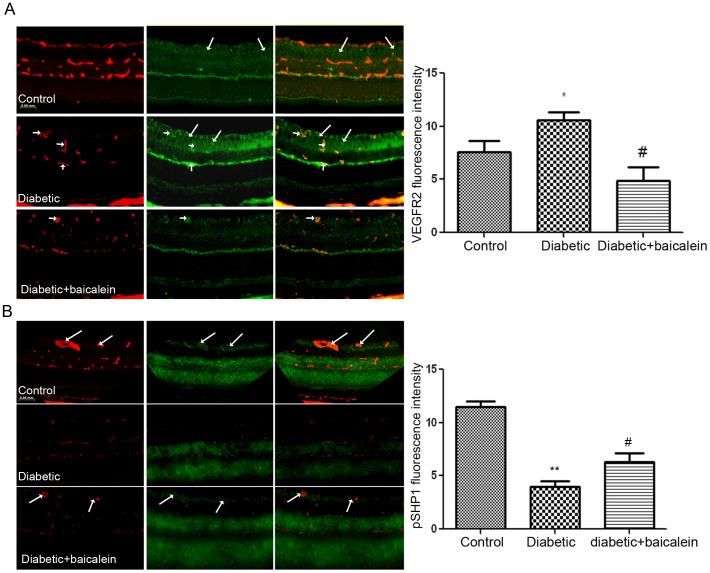
Immunofluorescence reaction of pVEGF-R2 and pSHP1 in retinal sections. There was marked increase in the pVEGF immunoreactivity in retinal sections from diabetic mice in comparison to the control. This was prevented by baicalein treatment (A). Note that pVEGF-R2 expression was localized mainly in Muller cells in the control (long arrows), however the reaction was also increased in retinal vessels during diabetes (short arrows) * P<0.05 vs control and # P<0.05 vs baicalein treated group (n = 5). pSHP immunolocalization (B) revealed marked decrease in diabetes which was restored by baicalein treatment. Note that pSHP is expressed in different retinal layers and also in relation to retinal vessels (arrows) in normal retina. In diabetes the decrease in pSHP occurred mainly in inner retina including retinal vessels. * P<0.05 vs control and # P<0.05 vs baicalein treated group (n = 5).

We also tested whether baicalein treatment impacts the levels of pVEGF-R2 in diabetic retina. The level of pVEGF-R2 was significantly higher in retinal section of diabetic mice in comparison with the control ones (10.6+0.7 vs 7.6+0.9). Administration of baicalein to diabetic mice prevented the increase in the level of pVEGF-R2 (4.8+1.3 vs 10.6+0.7) (Fig. 10A).

Since PTPs play important role in regulating the activity of VEGF-R2 we also evaluated the impact of baicalein treatment on the levels of pSHP1 using immunofluorescence reaction in retinal sections of different experimental groups. There was marked decrease in the levels of pSHP1 in diabetic mice in comparison to the control (4.1+0.5 vs 11.5+0.5). However the decrease in retinal pSHP1 by diabetes was significantly lesser in baicalein treated group (6.4+0.8) (Fig. 10B).

## Discussion

The present study investigated a potential molecular mechanism of the early inflammatory response in DR and mainly focused on the interaction between 12/15-LOX and NADPH oxidase in mediating retinal endothelial cell barrier dysfunction. Our major findings are, 1) Lipid metabolites of 12/15-LOX reduced TER and increased FITC-dextran flux through confluent monolayer of cultured REC, 2) Pro-permeability effect of 12/15-LOX metabolites was associated with reduced level of the TJP, ZO-1 and increased ROS generation and NOX2 expression, 3) NADPH oxidase inhibitors prevented the 12/15-LOX -mediated REC hyperpermeability, 4) Pharmacological inhibition of 12/15-LOX reduced the levels of inflammatory molecules, ROS generation and expression of NOX2 and pVEGF-R2 and restored the levels of ZO-1 and pSHP1 expression in retina of diabetic mice, 5) Activation of VEGF-R2 in REC by 12/15-LOX metabolites and prevention of HETE-induced REC permeability by VEGF-R2 inhibition.

12/15-LOX metabolizes arachidonic acid to produce 12- and 15-HPETEs and 12- and 15-HETE [37]. In humans, leukocyte type 12-LOX and reticulocyte-type 15-LOX-1 form similar metabolites, 12- and 15-HETE from common substrates and thus often referred to as 12/15-LOX s [38], [39]. Mice do not express 15-LOX and only express the leukocyte-derived 12-LOX which is also referred to as 12/15-LOX [40]. Accumulating evidence indicates that lipid products of 12/15-LOX are involved in the pathogenesis of retinal microvascular dysfunction during DR. Our recent studies indicated that 12/15-LOX plays a role in the development of pathological retinal NV. There was marked increase in 12-, 15- and 5-HETE in vitreous of PDR patients and in retina of mouse model of OIR. Additionally, retinal NV was reduced in mice lacking 12/15-LOX or treated with baicalein [10]. These findings suggested a role for 12/15-LOX in late stage of DR. Here, we investigated whether 12/15-LOX also contributes to the early inflammatory response as shown by increased levels of inflammatory mediators and vascular permeability during DR. For this purpose, we tested the impact of 12- and 15-HETE on REC endothelial cell barrier function by measuring the changes in the TER and FITC-dextran flux through a confluent REC monolayer. Our experiments demonstrated a significant increase in FITC-dextran flux and decrease in TER and ZO-1 expression by 12- and 15-HETE. These findings suggest that in addition to retinal NV, 12/15-LOX may also play a role in breakdown of BRB during DR. Although the role of 12/15-LOX in vascular permeability has not yet been well investigated particularly during DR, few recent studies reported pro-permeability role of 12/15-LOX in a variety of experimental models. For example, Yang et al. [17] demonstrated significant decrease of retinal vascular permeability in diabetic rats by baicalein. Furthermore, Cui et al. also reported marked decrease in brain edema by baicalein in a rat model of stroke [41] and attributed the preserving effect of atorvastatin on blood brain barrier (BBB) to the downregulation of 12/15-LOX in brain tissue [41]. Consistent with these findings, eliminating 12/15-LOX attenuated lung vascular permeability in a mouse model of acute lung injury [42] and inhibiting 12/15-LOX by N-benzyl-N-hydroxy-5-phenylpentana-mide (BHPP) reduced albuminuria in a model of diabetic nephropathy [43]. Taken together our and other recent studies suggest that 12/15-LOX metabolites are involved in mediating endothelial barrier disruption in various disease models including DR. However, the underlying mechanism is still not well-defined. Our current study showed that NADPH-oxidase is a potential mediator of the 12/15-LOX pro-permeability effect. Our experiments showed that NADPH oxidase inhibitors, apocynin and DPI preserved REC barrier function in HETE-treated REC. The effect of NADPH oxidase inhibitors was comparable to the ROS scavenger NAC suggesting that NADPH oxidase is involved in HETE-induced REC barrier dysfunction. The role of NADPH oxidase in the early inflammatory responses, leukostasis and permeability has been shown in our previous studies [22], [23]. In addition, inhibition of NADPH oxidase, has been shown by us and others to contribute to the vascular protective effects of statin therapy in DR [22], [24]. NADPH oxidase [44], activation of NFκB [45] and P38 MAP kinase [44], [46] are suggested mechanisms linking 12-LOX to diabetes-induced vascular complications such as nephropathy and atherosclerosis. Metabolites of LOXs can induce NADPH oxidase to stimulate ROS production [47], [48] suggesting the existence of an inter-connected signaling between LOXs and NADPH oxidase. Although the physiological relevance of the 12/15-LOX and NADPH oxidase pathways has yet to be clearly established, several researchers have suggested that 12/15-LOX acts upstream of the NADPH oxidase pathways [44], [49]. However, the exact mechanism by which NADPH oxidase-derived ROS mediate the pro-permeability effect 12/15-LOX lipid metabolites remains to be clarified. Our recent study showed that 12/15-LOX elicits its pro-angiogenic effect in retina probably through disrupting the VEGF/PEDF delicate balance in retinal glial cells by increasing expression of VEGF and downregulating PEDF. While our previous study showed that VEGF upregulation by 12-HETE was limited to glial cells, here we tested if HETE also impact VEGF-R2 signal pathways in REC. In particular, NADPH oxidase has been shown to modulate VEGF signaling pathway in endothelial cells via activating VEGF-R2 [50], [51]. Therefore, we also tested the impact of the 12/15-LOX metabolite 12-HETE on VEGF-R2 in REC as well as the impact of VEGF-R2 inhibition on the 12-HETE-induced REC hyperpermeability. Interestingly, we noticed a significant increase in the level of pVEGFR2 by 12-HETE and significant reduction in the12-HETE-induced REC hyperpermeability by the VEGF-R2 inhibition suggesting VEGF-R2 as a potential target for 12/15-LOX lipid metabolites which links 12/15-LOX activation to the REC barrier dysfunction during DR. Consistent with the *in vitro* findings, we also noticed significant abrogation in the pVEGF-R2 levels in retinal sections of the baicalein treated diabetic mice in comparison to the non-treated diabetic mice. Taken together our *in vitro* and *in vivo* findings indicate that VEGF-R2 signaling is involved in the permeability effect of 12/15-LOX -derived lipid metabolites.

Protein tyrosine phosphatases, a negative regulator of tyrosine kinase, are redox sensitive thiol-containing cysteine, which become inactive by oxidation [52], [53]. Several studies have shown role of PTPs in maintaining endothelial barrier function [54], [55] which probably due to inhibition of VEGF/VEGF-R2 signal pathway [56]. Consistent with this notion our data suggest that inhibition of PTP by NADPH-derived ROS might contribute to the increased phosphorylation of VEGF-R2, which in turn increased REC permeability by the 12/15-LOX metabolites.

Our *in vivo* experiments on akita mice as a model of DR confirmed the potential role for 12/15-LOX in the development of the early inflammatory responses during DR. Previous study by Yang et al. [17] reported that inhibition of 12/15-LOX with baicalein prevented the retinal microvascular changes in diabetic rats. Consistent with Yang et al., findings [17], our data showed a significant decrease in levels of the inflammatory mediators ICAM-1, VCAM-1 and IL-6 and restoration of ZO-1 expression in diabetic mice treated with baicalein. Effect of baicalein was also associated with reduced 12- and 15-HETE production, ROS generation and NOX2 expression. Our data suggest a correlation between the inhibitory effect of baicalein on 12/15-LOX and the decreased inflammatory reaction, oxidative stress and vascular permeability as shown by us and Yang et al. [17].

Taken together our previous [10], current findings as well as the findings of Yang et al., [17] we believe that inhibition of 12/15-LOX could be a novel therapeutic strategy to prevent the development of microvascular dysfunction during DR. However further studies are necessary to validate the therapeutic effects versus side effects as well as its benefit over direct inhibition of VEGF-R2.Furthermore, mode of delivery of 12/15-LOX inhibitors could be also an area of future research to evaluate the impact of systemic versus local intraocular delivery. There is also now considerable evidence supporting the proinflammatory role of 12-LOX via promoting leukocyte/endothelial interaction in NFkb and ICAM-1-dependent pathway [45]. Therefore, targeting 12/15-LOX may interrupt not only VEGF-R2 signaling pathway but also other important pathways required for the early inflammatory response during DR.

**Figure 11:**
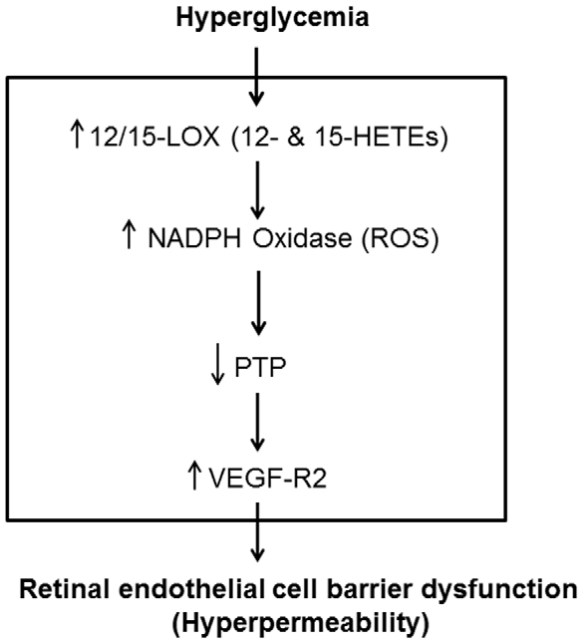
Proposed cascade of events following activation of 12/15-LOX by hyperglycemia. Lipid metabolites of 12/15-LOX (12- and 15-HETE) activate vascular NADPH oxidase leading to overproduction of reactive oxygen species (ROS). Generation of ROS suppresses the activity of protein tyrosine phosphatases with subsequent activation of VEGF-R2 signal pathway and disruption of retinal endothelial cell barrier.

In conclusion, our data suggest that 12/15-LOX contributes to the early inflammatory response and breakdown of BRB during DR. The underlying mechanism includes activation of NADPH oxidase, inactivation of PTP and subsequent activation of VEGF-R2 signal pathway (Figure 11). The current study proposes 12/15-LOX pathway as a potential therapeutic target to prevent the development and progression of DR.
